# Thoracic ultrasound: a review of the state-of-the-art

**DOI:** 10.36416/1806-3756/e20230395

**Published:** 2024-09-16

**Authors:** Philippe de Figueiredo Braga Colares, Thiago Thomaz Mafort, Felipe Marquesini Sanches, Laura Braga Monnerat, Carlos Augusto Metidieri Menegozzo, Alessandro Wasum Mariani

**Affiliations:** 1. Divisão de Pneumologia, Departamento de Cardiopneumologia, Instituto do Coração - InCor - Hospital das Clinicas Faculdade de Medicina, Universidade de São Paulo, São Paulo (SP) Brasil.; 2. Hospital de Base de São Jose do Rio Preto, Faculdade de Medicina de São Jose do Rio Preto, São Jose do Rio Preto (SP) Brasil.; 3. Departamento de Pneumologia, Faculdade de Ciências Médicas, Universidade do Estado do Rio de Janeiro - UERJ - Rio de Janeiro (RJ) Brasil.; 4. Divisão de Cirurgia Geral e Trauma, Departamento de Cirurgia, Faculdade de Medicina, Universidade de São Paulo, São Paulo (SP) Brasil.; 5. Divisão de Cirurgia Torácica, Departamento de Cardiopneumologia, Instituto do Coração, Hospital das Clínicas, Faculdade de Medicina, Universidade de São Paulo, São Paulo (SP) Brasil.

**Keywords:** Thorax/diagnostic imaging, Ultrasonography/methods, Point-of-care testing, Lung/diagnostic imaging

## Abstract

Thoracic ultrasound (TUS) is a tool that has become increasingly essential in the daily practice of thoracic medicine. Driven by the need to assess patients during the COVID-19 pandemic, there has been an increase in the use of point-of-care TUS, which has demonstrated several benefits, either as a complement to clinical decision-making for diagnosis or as a real-time guide for procedures, whether as a predictor or measure of treatment response. Here, we present a review of TUS, based on the most recent scientific evidence, from equipment and techniques to the fundamentals of pulmonary ultrasound, describing normal and pathological findings, as well as focusing on the management of lung disease and guidance for invasive thoracic procedures at the bedside. Finally, we highlight areas of perspective and potential lines of research to maintain interest in this valuable tool, in order to improve the diagnostic process and expand the treatment arsenal.

## INTRODUCTION

Thoracic ultrasound (TUS) has become increasingly essential in the daily practice of pulmonologists and thoracic surgeons.^1^ ^-^ ^3^ For many years, the use of ultrasound was seen as insufficient for the evaluation of pulmonary diseases, being practically restricted to the ICU and emergency department (ED).^1^ Recently, prompted by the need to assess patients during the COVID-19 pandemic, point-of-care TUS has gained ground in clinical practice and has demonstrated several benefits, either as a complement to clinical decision-making for diagnosis or as a real-time guide for procedures, whether as a predictor or measure of treatment response.^1^ ^-^ ^4^

Faced with the growing demand, we prepared a review of TUS, from equipment and techniques to the fundamentals of pulmonary ultrasound, based on the most recent scientific evidence, describing normal and pathological findings, focusing on patient management with pulmonary pathologies, as well as on guidance for invasive thoracic procedures at the bedside. Finally, we highlight areas of perspective and potential lines of research to maintain interest in this valuable tool in order to improve our diagnostic capability and expand our treatment arsenal.

## EQUIPMENT AND TECHNIQUES

Various protocols,^1^ ^-^ ^5^ techniques, and types of equipment have been described and validated for use in TUS, which can be performed with practically any ultrasound system capable of two-dimensional scanning, with conventional brightness (B)-mode, although options such as motion (M)-mode and color flow Doppler can also be utilized. 

The ideal probe will depend on the clinical setting and suspected diagnosis.^1^ ^-^ ^3^ A low-frequency (5-2 MHz) curvilinear probe allows visualization of deeper structures and acceptable visualization of the pleural line, ideal for evaluating deeper pathologies, such as pleural effusion (PE) and diseases of the lung parenchyma.^3^ High-frequency (14-6 MHz) linear probes generate highly detailed images of superficial structures, which include the intercostal musculature, rib margins, and pleural anatomy.^3^

 The patient can be examined in the supine position or in a sitting position (from the back), and the probe can be positioned in the longitudinal or transverse (intercostal) orientation, although it should be nearly perpendicular to the skin surface or pleural line.^1^ ^-^ ^3^

## NORMAL FINDINGS ON TUS

Because of the principles of ultrasound propagation in aerated structures, TUS relies mainly on the interpretation of artifacts.^1^ ^-^ ^4^ Recognizing the role of artifacts, especially the A-lines and B-lines, in normal and abnormal pathologies is critical to understanding TUS.^4^ ^,^ ^5^

 The A-lines are reverberation artifacts that appear as parallel echogenic lines arranged below the pleural line and repeated at regular intervals, equidistant from the skin to the pleural surface.^4^ In turn, the B-lines emanate perpendicularly from the pleural surface, extend to the depth of the image without decreasing in intensity, and move synchronously with lung sliding (Chart 1). Their characteristic feature is that they obscure A-lines and, in isolation, can also be seen in the aerated lung.^4^

**Chart 1:** Thoracic ultrasound profiles.

Syndrome	Ultrasound signs
Aerated lung (normal findings)	Predominant anterior bilateral A-lines (batwing sign), associated with lung sliding (seashore sign), and the curtain sign, at the costophrenic recess.
Interstitial syndrome	Diffuse bilateral anterior B-lines (at least three or more B-lines in 2 or more thoracic regions) associated with lung sliding.
Pleural effusion	Anechoic fluid or homogeneously echogenic fluid between the pleural leaflets, with or without floating debris, septations, or other structures within the effusion.
Pneumothorax	Absent anterior lung sliding (barcode or stratosphere sign), absent anterior B-lines and present lung point.
Pneumonia	Predominant anterior B-lines on one side or in one thoracic region, with predominant anterior A-lines on the other; or presence of alveolar (tissue-like) consolidation

**Figure 1:**
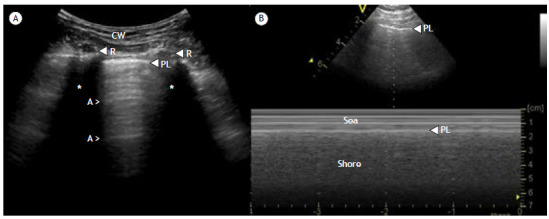
Aerated lung in two-dimensional (2D) mode and corresponding motion (M)-mode image. a) Normal findings: the muscles, fascia, and other soft tissues of the chest wall (CW) are in the upper part of the image. The surfaces of the ribs (R) can be seen as two horizontal hyperechoic, white lines with posterior acoustic shadowing (*). The pleural line (PL) is located just below the ribs. The lung tissue is filled with air and therefore cannot be seen. Consequently, the area that can be seen below the PL is not the lung tissue but artifacts, represented by the A-lines (A). The ribs resemble the wings of a bat, whereas the PL mimics the body of the bat, a pattern known as the batwing sign. b) Normal M-mode findings: the M-mode line can be seen running vertically through the PL at the top of the image. In the corresponding M-mode image, the PL is seen as a hyperechoic line placed at the same distance from the transducer as can be seen in the 2D image. Note the seashore sign, which is so named because, in M-mode, the static structures of the CW can appear as horizontal lines above the PL (representing the sea) and, in the presence of lung sliding, the area below the PL will have a grainy appearance (representing the shore).

Normal TUS findings include visualization of the intercostal musculature, rib shadows, and the pleural line, together with the presence of A-lines, giving rise to the batwing sign.^1^ ^-^ ^4^ The natural motion of the visceral and parietal pleura results in a phenomenon known as lung sliding, seen in M-mode as the seashore sign (Figure 1). Finally, at the costophrenic recess, the overlap of the aerated lung onto the abdomen creates a demarcated leading edge of the lung air artifact, giving the impression of a lung curtain, known as the curtain sign.

## ALVEOLAR-INTERSTITIAL SYNDROME

Alveolar-Interstitial syndrome (AIS) describes several conditions characterized by diffuse interstitial involvement and impaired gas exchange across the alveolar-capillary membrane, potentially leading to respiratory failure.^5^ ^,^ ^6^ Causes can be acute or chronic, including interstitial lung disease, ARDS, and acute pulmonary edema. Pulmonary ultrasound has emerged as a noninvasive tool with the potential to detect AIS at the bedside, with a sensitivity of 85.7% and specificity of 97.7%.^6^

**Figure 2:**
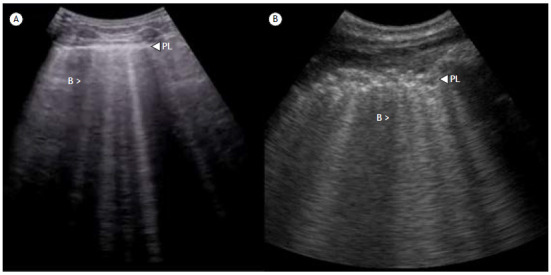
a) Multiple B-lines (B) can be seen as vertical, hyperechoic lines originating from the pleural line (PL) and stretching all the way to the bottom of the two-dimensional brightness (B)-mode image. b) Idiopathic pulmonary fibrosis: thoracic ultrasound image of the lower lobe of a patient diagnosed with idiopathic pulmonary fibrosis. Multiple B-lines are present, and the PL appears severely thickened and fragmented.

The sonographic diagnosis of AIS is based on the presence and quantification of B-lines (Figure 2), also known as comet tail artifacts.^7^ The most widely used definition of AIS is the presence of at least three or more B-lines in the longitudinal plane in two or more anterior or lateral bilateral thoracic regions.^1^ While three to four B-lines correlate better with interlobular septal thickening, five or more correlate with areas of ground-glass opacity and indicate a more severe interstitial syndrome.^7^

**Chart 2:**
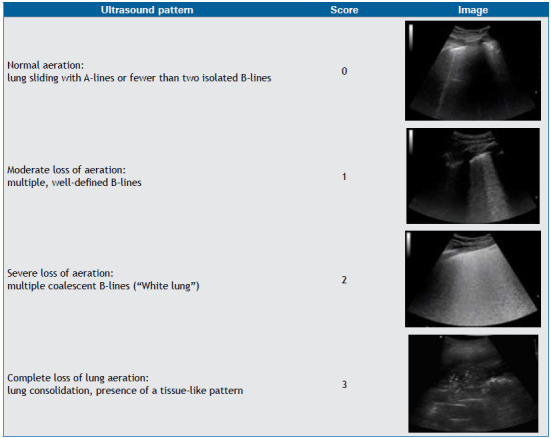
Lung ultrasound score.

The quantification of B-lines demonstrates a positive linear correlation with extravascular lung water assessed by radiological scores, transpulmonary thermodilution methods, and Wedge pressure by right heart catheterization.^7^ ^,^ ^8^ Loss of aeration can be quantified by using validated scores like the Lung Ultrasound Score, which evaluates six lung regions on each side, assigning a score of 0-3 to each region based on aeration (Chart 2). The global lung ultrasound score is the sum of the regional scores and therefore ranges from 0 to 36.^9^

Lung ultrasound score assessment. Six lung zones of interest are examined on each side, delineated by a parasternal line, anterior axillary line, posterior axillary line, and paravertebral line. Each lung region is carefully examined in the longitudinal plane, and each intercostal space present in the region is reviewed in the transverse plane. A semiquantitative score ranging from 0 to 3 is performed according to the lung ultrasound findings in each zone, graded as follows: 0 = normal aeration; 1 = moderate loss of aeration (interstitial syndrome, defined by multiple spaced B-lines, localized pulmonary edema, characterized by coalescent B-lines in less than 50% of the intercostal space examined in the transverse plane, or subpleural consolidations); 2 = severe loss of aeration (alveolar edema, defined by diffuse coalescent B-lines occupying the entire intercostal space); and 3 = complete loss of lung aeration (lung consolidation defined as a tissue pattern, with or without air bronchogram). The global lung ultrasound score is calculated as the sum of the 12 regional scores (ranging from 0 to 36).

Pulmonary ultrasound stands out in diagnosing interstitial syndromes within and outside the ICU and ED settings. It is a highly accurate, noninvasive tool for diagnosing acute decompensated heart failure in the ED.^10^ ^,^ ^11^ In cases of interstitial lung disease, it has the potential to serve as a screening tool, demonstrating sensitivity comparable to that of HRCT, especially in patients with systemic sclerosis.^12^ ^,^ ^13^ In addition, for patients with ARDS, the identification of bilateral lung opacities by ultrasound has been incorporated into the new diagnostic criteria.^14^ Despite the presumed capability of ultrasound to assess focal and diffuse lung aeration loss and its potential to predict the response to recruitment maneuvers, that capability has yet to be definitively confirmed.^15^

## CHEST WALL

The evaluation of the chest wall by TUS includes the analysis of subcutaneous tissue, muscle groups, and ribs. Because those are superficial structures, a high-frequency (14-6 MHz) linear transducer is typically used. 

The main indications for the use of this method are for the investigation of localized pain, palpable alterations on physical examination, liquid collections in the chest wall (bruises, postoperative seromas, and abscesses), and solid lesions (nodulations and tumors), as well as for clarifying findings from other imaging modalities. Chest wall TUS can also be used in order to guide punctures and biopsies.^16^

 Yet another use is in the evaluation of lytic or blastic bone lesions and rib fractures, in which a loss of linearity of the cortical layer can be observed.^17^

## VISCERAL AND PARIETAL PLEURA

Differentiating between the parietal and visceral pleura can be challenging because each leaflet typically measures only approximately 0.2 mm, which may exceed the detection power of ultrasonography. Given that the pleura is a relatively superficial structure, it can be examined by TUS, usually through the intercostal window, visualized as a hyperechoic line below the ribs.

**Figure 3:**
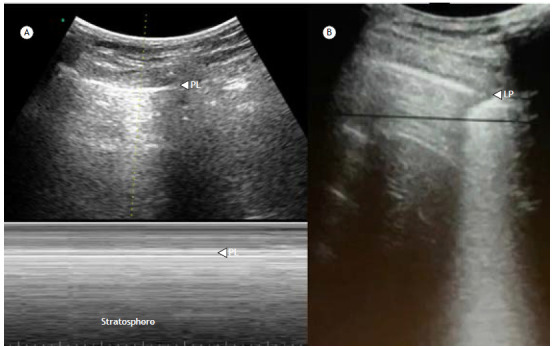
Pneumothorax. a) Motion (M)-mode findings in pneumothorax: if lung sliding and the lung pulse are absent, there will be no change in the area below the pleural line in the two-dimensional (2D)/brightness (B)-mode image. In M-mode, horizontal lines will be seen above and below the pleural line (PL), and the seashore sign can no longer be identified. The M-mode pattern has been described as resembling a barcode or a stratosphere and is therefore known as the barcode sign or stratosphere sign, which can be seen when a pneumothorax is present, as well as in conditions in which lung sliding and the lung pulse are absent (e.g., pleural adhesions). b) 2D-mode showing the lung point (LP). The LP is an ultrasonographic sign that is used in order to locate the junction between the pneumothorax and the area with no air between the visceral and parietal pleural and refers to a pattern of repeated transitions between no lung sliding or B-lines (pneumothorax) into a demonstrable area of lung sliding.

In healthy individuals, in addition to identifying the pleural line, we can also see the leaflets sliding, either in the two-dimensional ultrasound mode (lung sliding) or confirmed by the seashore sign in M-mode. When there is no sliding, the M-mode generates the so-called barcode sign, also known as the stratosphere sign (Figure 3).^18^

## SOLID PLEURAL LESIONS

The pleural tissue can be involved in various malignant and benign processes. Diffuse pleural thickening is commonly associated with exudative PE, hemothorax, or empyema. Focal or circumscribed pleural thickening may correspond to inflammation (pleuritis) or malignant infiltration.^19^ ^,^ ^20^ These findings can help determine the need for a pleural procedure and the most appropriate site for such.

Pleural plaques associated with asbestosis can be identified by their elliptical hypoechoic aspect. Benign tumors (lipomas, fibromas, chondromas, neurinomas, and mixed forms) account for only 5% of neoplastic lesions in the pleura. On ultrasound, these tumors are typically round or oval and encapsulated, with a well-defined outline, and are hypoechoic or moderately echogenic. In general, lung mobility is preserved, and the tumors might be vascularized, which can be confirmed in Doppler mode.^19^

 However, malignant pleural tumors (metastases, lung tumor with extension, and malignant mesothelioma) are more common. Signs of malignancy include irregular thickening or nodularity of the pleura, with a heterogeneous ultrasound pattern, associated with PE and infiltration of adjacent structures. In malignant mesothelioma, pleural thickening usually exceeds 10 mm and can be focal or diffuse. Pleural metastases typically have a hypoechoic, homogeneous appearance with an oval or irregular outline. In lung tumors with transpleural extension, pleural sliding tends to be compromised, with invasion of the chest wall and the ribs occasionally being observed, which represents a reliable sign of lung injury from direct tumor extension.^20^

## PLEURAL EFFUSION

Given the current evidence, TUS may be considered the imaging method of choice for the initial assessment of PE, being employed to assess pleural fluid volume and character, as an auxiliary method in the search for the etiology, and as a method for guiding thoracentesis or chest drainage.^21^

With TUS, we can identify much smaller fluid volumes (even as small as 20 mL) than with other modalities, particularly chest X-ray, and avoid many of the negative aspects of CT, because TUS can be performed in real time at the bedside, with very high spatial resolution.^2^ ^,^ ^22^ Although an accurate quantitative assessment of pleural fluid volume may be possible with TUS, the qualitative assessment is adequate for most clinical decision-making by categorizing the fluid volume as minimal, small, moderate, or large.

The addition of color Doppler may improve the assessment and the differentiation between fluid and pleural thickening. Solid pleural and peripheral lung lesions are generally hypoechoic and show no flow on color Doppler ultrasound, whereas pleural fluid may generate a colored flow pattern during respiratory or cardiac cycles, known as the fluid color sign.^22^

**Figure 4:**
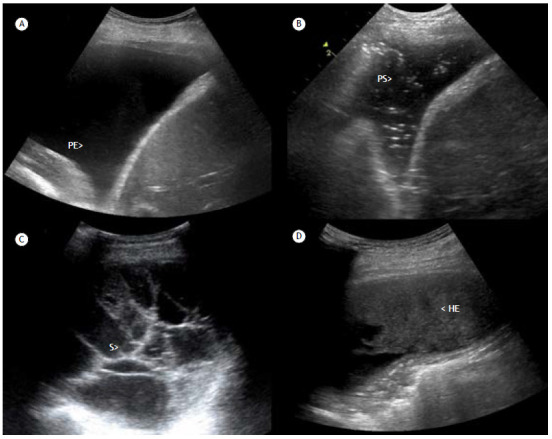
Pleural effusion and its varied presentations. a) Simple pleural effusion: a simple, anechoic, pleural effusion (PE) is present. There are no septations or visible structures floating within the effusion. b) Complex nonseptated PE: anechoic fluid with the presence of multiple hyperechoic punctuate foci representing floating debris within the effusion, also known as the plankton sign (PS). c) Complex septated PE: a complex septated PE is present, containing areas of anechoic fluid as well as several septa (S)/loculations. d) Homogeneously echogenic (HE) fluid: a combination of HE fluid with a stratification effect in the costophrenic recesses (the hematocrit sign) may suggest the presence of hemothorax.

According to its appearance on ultrasound (Figure 4), PE can be classified into four categories^19^:

- Simple PE-anechoic effusion, with no echo between the pleural leaflets; represents free fluid without the presence of septations or other structures within the fluid
- Complex nonseptated PE-anechoic fluid with multiple hyperechoic punctuate foci (swirling echoes) representing floating debris within the effusion, also known as the plankton sign; denotes greater liquid density, by the presence of cells, fibrin, or proteins, but without septa
- Complex septated PE-anechoic fluid with the presence of several septa, forming pockets (loculations) between the pleural leaflets
- Homogeneously echogenic PE-a homogeneous area with a hypoechoic structure; denotes a high-density liquid, such as pus or blood

This classification can help differentiate between exudates and transudates. Transudative PE, as a rule, will appear as a simple PE, although it is not a specific finding. Conversely, exudative PE will almost always appear as echogenicity or complexity. Although there is a strong correlation between the swirling echoes sign and exudative processes, that is also not a specific sign.

Other pleural changes that suggest exudates include pleural thickening and the presence of nodules (or tumors). Alterations in the lung parenchyma consistent with an infectious process (such as consolidation), when accompanied by PE, also suggest exudates.^19^ ^,^ ^20^

There are sonographic changes that also point to specific causes of PE. The presence of septations, loculations, or homogeneously echogenic fluid, in the clinical suspicion of infection, supports the possibility of complicated parapneumonic effusion or empyema and generally requires pleural drainage. The presence of bubbles in the pleural fluid, described as the suspended microbubble sign, is highly sensitive and specific for empyema.^2^ ^,^ ^22^

Homogenously echogenic effusions are most often due to hemothorax or empyema. The high cell count of a hemothorax creates a layering effect in the costophrenic recesses, known as the hematocrit sign. In the appropriate clinical context, a combination of the hematocrit sign and the plankton sign is suggestive of hemothorax.^2^ ^,^ ^22^

When there is pleural or diaphragmatic nodularity and thickening (mainly greater than 10 mm), we should consider the possibility of tumor involvement, which typically presents with the swirling echoes sign. The use of TUS also allows the evaluation of adjacent structures such as the diaphragm, soft tissues, bones, abdominal organs, and mediastinum, which can be helpful in diagnostic clarification.^23^

## PNEUMOTHORAX

For evaluating pneumothorax, TUS is useful because it is a noninvasive test that can be performed at the bedside, with good sensitivity and excellent specificity for rapid detection of this pathology.^24^ Four important sonographic signs indicate the presence of pneumothorax^25^ ^,^ ^26^:

- Absence of pleural sliding-The presence of air between the two pleural surfaces leads to the disappearance of pleural sliding. M-mode can be used to confirm the suspicion, on the basis of the characteristic barcode or stratosphere sign (Figure 3). It is noteworthy that pleural sliding may be absent in other conditions, such as pleurodesis, extensive pulmonary fibrosis, and reduced lung compliance.
- Presence of A-lines-These artifacts are present in normally aerated lungs but also in pneumothorax, in which case A-lines are visible but B-lines are not, a finding that has 100% sensitivity for diagnosing pneumothorax. Combining this sign with the absence of lung sliding also has high specificity.
- Absence of the lung pulse-The lung pulse consists of a vertical movement of the pleural line synchronous with the heartbeat (observed in M-mode). In pneumothorax, intrapleural air prevents the transmission of the lung pulse to the parietal pleura, with the consequent absence of lung sliding and of the lung pulse. In contrast, the presence of the lung pulse rules out pneumothorax.
- Presence of the lung point-The lung point is defined as the transition between the area of normality and that of a pneumothorax. After the absence of pleural sliding and the presence of A-lines have been confirmed, the point where the sliding begins should be identified. That will be the edge of the pneumothorax. The lung point can also be identified in M-mode, in which the barcode sign is followed by the seashore sign. This sign only occurs in pneumothorax, having 100% specificity.

The presence of the lung point can also correlate with the pneumothorax volume. When located medial to the midaxillary line, it indicates 15% pulmonary collapse, suggesting conservative management, whereas when it is lateral to the midaxillary line it represents significant collapse and indicates a need for drainage. However, this quantification strategy has been validated only in trauma patients, and caution should therefore be exercised when extrapolating to other populations.^27^

## PNEUMONIA

In the initial assessment and follow-up of patients with a suspected respiratory infection, TUS has increasingly been used as an imaging method, with better sensitivity and accuracy than chest X-ray.^28^ One limitation of the use of TUS in this context, however, is that it cannot assess parenchymal changes that are not in the subpleural region. Nevertheless, most infections that affect the lung parenchyma also affect the subpleural region and decrease lung aeration. Therefore, it is possible to identify specific artifacts that correlate with pathological changes caused by pneumonia.^18^

The pattern most often encountered in pneumonia is focal B-lines, which correlate with ground-glass areas and incomplete filling of the alveoli. An asymmetrical pattern typically occurs, with B-lines in each region and A-lines in other regions (including the other hemithorax). With TUS, subpleural consolidation can be identified with excellent sensitivity. Other potential alterations include pleural irregularity and the absence of pleural sliding. In addition, TUS is quite useful for identifying associated PE and possible complications such as empyema and lung abscess.^29^

The recent COVID-19 pandemic has shown the great utility of TUS in patient evaluation, either as an auxiliary method in screening for respiratory symptoms or in the monitoring of hospitalized patients. Several studies have shown that the ultrasonographic alterations observed in the parenchyma and the pleural surface on TUS correlate with those observed on chest CT. In addition, the degree of parenchymal involvement, characterized by decreased lung aeration, seen on TUS has been shown to be associated with symptom worsening and mortality.^30^

The portability of TUS was another great advantage during the pandemic, allowing its use in diverse scenarios. In addition, in intensive care settings, it proved to be an excellent tool for monitoring patients, including those on mechanical ventilation, allowing the assessment of various parameters, such as lung aeration after alveolar recruitment maneuvers.^31^

## DIAPHRAGM

Another use for TUS is in the direct evaluation of the mobility and contraction of the diaphragm. During the assessment of diaphragmatic mobility, a low-frequency convex transducer is used and the image is usually obtained through the hepatic window in the right subcostal region. On the left side, the splenic window can be used, although visualization of the diaphragm is usually more complex and measurements tend to be less reproducible.

Mobility is best assessed in M-mode, and measurements can be made in tidal volume, maximal inspiration, maximal expiration, and during the sniff test. The transducer must be positioned between the anterior axillary and midclavicular lines in the cranial-dorsal direction. The measurements are usually more straightforward and more reproducible when the patient is in the supine position.

Mobility of less than 10 mm is considered indicative of severe diaphragmatic dysfunction. Another marker of dysfunction is paradoxical movement of the muscle, usually seen in the sniff maneuver when the diaphragm insinuates into the thoracic cavity during rapid inspiration.^32^ Studies evaluating normality values for diaphragmatic mobility have produced discrepant results. In a recent study of 757 healthy individuals, the following results were obtained for diaphragmatic mobility^33^:

- Men during tidal volume: 2.37 ± 0.53 cm
- Men during deep breathing: 5.74 ± 1.26 cm
- Women during tidal volume: 2.22 ± 0.54 cm
- Women during deep breathing: 5.20 ± 1.19 cm

Muscle contraction can be evaluated in the apposition zone, an anatomical region located at the transition between the thorax and abdomen, in which the diaphragm is covered by the pleura (in its portion closest to the thoracic wall) and by the peritoneum (in its portion closest to the abdominal cavity). For this evaluation, a high-frequency linear transducer is used, making it possible to visualize muscle contraction and to determine the thickening fraction, which is calculated with the following formula:

Tins−Tex∕Tex×100

where *Tins* is the maximum thickness during inspiration and *Tex* is the maximum thickness during expiration. Muscle contraction is considered adequate when the thickening fraction exceeds 20% (or 30%, according to some authors.^32^ ^,^ ^33^

Measurements of diaphragmatic mobility and the thickening fraction have both been used in various settings and inform clinical reasoning in outpatient and intensive care settings. The findings in the evaluation of the diaphragm even correlate with successful weaning from mechanical ventilation. Some data show a correlation between the degree of hyperinflation and diaphragmatic impairment in patients with COPD. Another area that has gained ground is the evaluation of diaphragmatic paresis and paralysis in neuromuscular diseases. In such cases, the TUS findings, in addition to the clinical data, provide predictive information about the progression to respiratory failure.^32^ ^,^ ^34^

## ULTRASOUND-GUIDED PROCEDURES

### Endotracheal intubation

Ultrasound is a powerful adjunct in endotracheal intubation and can be used to exclude esophageal intubation and to check tube selectivity. These evaluations can be performed with low- or high-frequency transducers. The latter should provide more detailed images given that both of the sonographic applications discussed target superficial tissues. 

Classic methods to detect endotracheal intubation are based on ventilation, with capnography being the gold standard. However, capnography is not available in all EDs and ICUs. In practice, confirmation of a successful procedure is traditionally performed by lung auscultation during ventilation. Thus, if the intubation was inadvertently esophageal, there is a risk of gastric distention and bronchial aspiration during confirmation of the endotracheal tube placement.

When compared with the gold standard (capnography), ultrasound has been shown to display high accuracy in rapidly identifying esophageal intubation, with no risk of insufflation. Two meta-analyses confirmed these findings by showing ultrasound to have a sensitivity of 93-98% and a specificity of 97-98% for that purpose.^35^ ^,^ ^36^

**Figure 5:**
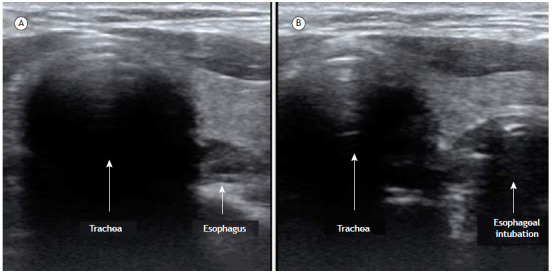
Endotracheal intubation. a) Axial image of the cervical region obtained with a linear transducer identifying the trachea and esophagus. b) The same ultrasound window shows the appearance of the esophagus with an orotracheal tube inside, illustrating esophageal intubation (double tract sign).

The physician can use ultrasound in real time or after the intubation (Figure 5). Real-time ultrasound evaluations require two sonographers. One will execute the intubation itself while the other positions the ultrasound probe on the neck of the patient (dynamic evaluation). A rapid ultrasound examination of the trachea during the procedure excludes esophageal intubation in real time.^37^ However, the same sonographer can evaluate the position of the tube shortly after the procedure (static evaluation).

While selectivity can be appreciated with lung auscultation, this method has lower diagnostic accuracy. Ultrasound can detect orotracheal tube selectivity by identifying the position of the endotracheal balloon or, more often, by visualizing bilateral lung sliding. A prospective study compared ultrasound and auscultation in terms of their accuracy in excluding tube selectivity. By comparing both methods with the gold standard (fiberoptic bronchoscopy), the study demonstrated that the accuracy of ultrasound and lung auscultation was 95% and 62%, respectively, confirming the superiority of the sonographic evaluation.^38^

### Percutaneous tracheostomy

Percutaneous tracheostomy is currently the technique of choice for facilitating mechanical ventilation in critically ill patients. Studies have shown that percutaneous tracheostomy has lower costs and lower complication rates than does conventional tracheostomy.^39^

Although percutaneous tracheostomy is most often performed with the aid of bronchoscopy, ultrasound guidance has been increasingly used. In comparison with the technique guided by anatomical landmarks, ultrasound-guided percutaneous tracheostomy results in a better choice of puncture site, shorter procedure time, fewer punctures, and fewer complications.^40^ In comparison with the technique guided by bronchoscopy, the ultrasound-guided procedure has the advantage of evaluating anterior cervical structures, although it is limited by not providing visualization of the posterior wall of the trachea. However, recent studies have shown that both techniques have a similar safety profile.^41^ ^,^ ^42^

To undergo ultrasound-guided percutaneous tracheostomy, patients should ideally be positioned with cervical hyperextension. The high-frequency linear probe is the best choice for the procedure because it yields detailed images of the superficial structures. The most common techniques to perform percutaneous tracheostomy were described by Ciagla et al.^43^ and Griggs et al.^44^

**Figure 6:**
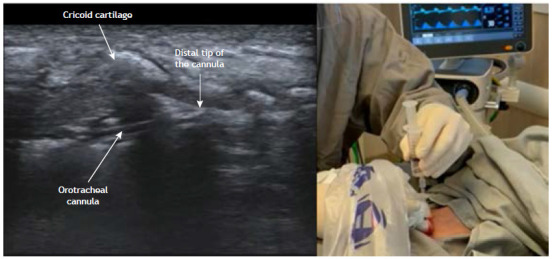
Percutaneous tracheostomy. a) Sagittal view of the trachea showing the orotracheal cannula represented by a double hyperechoic line with acoustic shadowing. The tip of the endotracheal tube is ideally positioned under the first tracheal ring to ensure a clear path for the tracheal puncture while reducing the risk of accidental extubation. b) Repositioning of the endotracheal tube prior to midline perpendicular tracheal puncture is performed under ultrasound guidance, thus reducing the risk of complications. The patient should be placed in the supine position.

Although some authors have described various preparatory and technical steps associated with increased safety of the procedure,^45^ such technical details were beyond the scope of this review. In summary, after identifying a safe site for puncture, the endotracheal tube is pulled, under ultrasound guidance, to a point that allows insertion of the needle into the trachea without accidental puncture of the tube. Tracheal puncture is performed in the midline with ultrasound guidance (Figure 6). The protocol then proceeds to dilation and placement of the tracheal tube, followed by confirmation of adequate ventilation. Ultrasound allows visualization of cervical structures, identification of blood vessels in the path of the puncture, guided traction of the tube, centralization of the tracheal puncture, and identification of immediate complications of the procedure.

### Thoracentesis and tube thoracostomy

Ultrasound-guided percutaneous drainage of intrathoracic and pleural collections has several advantages over blind (i.e., unguided) procedures. First, as previously reported, ultrasound can help differentiate between simple and complicated PE, which can facilitate the choice between thoracentesis and tube thoracostomy.

For determining the optimal puncture site in the chest wall, ultrasound enables the sonographer to identify relevant structures, such as the diaphragm, vessels, and nerves, that might be in the drainage tract. It also allows real-time visualization of the collection and the needle tip during its progression, improving the accuracy of the procedure and reducing the risk of complications such as pneumothorax and organ injury.^46^ ^,^ ^47^ In addition, ultrasound guidance can help to optimize drainage by identifying loculations or septations within the collection that may require specific needle positioning or redirection.^48^ With TUS, we can also monitor the drainage process by detecting changes in the size and location of the collection in real time, making adjustments to the needle position as needed. This real-time monitoring and adjustment can increase the efficiency of the procedure and minimize the need for repeated attempts or multiple punctures.

Image guidance is particularly useful in cases in which the collection is very small or is in a complex or challenging area, such as near the diaphragm. Guidance with TUS can increase the success rate of percutaneous drainage of such collections, although their localization often requires guidance by other imaging modalities for safety reasons.^49^

Thoracentesis guided by TUS can be performed through site marking or direct needle guidance.^50^ In the site marking method, the sonographer identifies the ideal puncture site under TUS guidance and marks it on the skin, then performs the thoracentesis without guidance. In this case, a change in patient position can cause fluid redistribution; therefore, the procedure should be performed immediately after marking the site. In the direct needle guidance method, the correct needle position is visualized and monitored in real time.

Albeit a common procedure, tube thoracostomy still has a reported complication rate of 14-25%, with complications ranging from those caused by incorrect drain placement to lethal iatrogenic injuries.^51^ The routine use of TUS can diminish these risks. Menegozzo et al.^52^ described a standardized protocol for ultrasound-guided pleural drainage in which the use of ultrasound is primarily aimed at reducing complications related to drain insertion, identifying a poorly positioned drain (in the subcutaneous tissue) early on and ruling out the presence of a vulnerable neurovascular bundle in the intercostal space. That protocol may be used with trocars (i.e., pigtail catheters) or with blunt dissection.

With TUS, which allows visualization of the diaphragm, some cases of diaphragmatic hernia can be identified, potentially reducing subdiaphragmatic insertions and organ injury during pleural drain insertion. Excluding subcutaneous placement at the end of the procedure allows quicker repositioning, reducing the potential negative implications of a malfunctioning drain, and identifying a vulnerable intercostal artery may reduce the incidence of vascular injuries and their complications.^51^ ^,^ ^52^

After routine patient preparation, the ultrasound sonographer assesses the regional anatomy to define the drain insertion site. This is done by observing diaphragmatic excursion and the intercostal space, excluding a vulnerable intercostal artery. The intercostal space that does not demonstrate diaphragmatic excursion is preferably used, in order to avoid diaphragmatic injuries. Local anesthesia can then be administered with the aid of ultrasound. The actual drain insertion follows the traditional technique.

**Figure 7:**
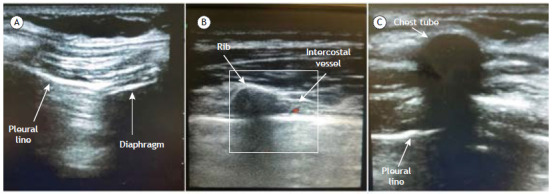
Tube thoracostomy. a) Brightness (B)-mode image, obtained with a linear probe, showing diaphragmatic excursion and a normal lung. The examiner evaluates the range of diaphragmatic excursion during a full cycle of ventilation. This enables the examiner to choose the lowest site for tube insertion while avoiding injury to the diaphragm. If the previously selected site exhibits diaphragmatic movement, a more cranial intercostal space must be scanned. b) Visualization of intercostal vessels using Doppler ultrasound. Once the examiner finds a suitable intercostal space, the insertion site should be scanned with Color Doppler. The intercostal artery most commonly lies on the upper third of the intercostal space. The entire intercostal space should be scanned in order to make sure that there is no blood flow along the insertion path. c) Confirmation of correct positioning of the drain. Axial view of a large-bore chest tube, showing the characteristic hyperechoic arc over a black circle with posterior acoustic shadowing, seen along the subcutaneous plane. Following that image along the drainage site, one should see the drain deepening toward the pleural line.

If the procedure is a thoracentesis, ultrasound should be used after the fluid drainage to check for residual collections, to verify lung expansion, and to identify complications such as hemothorax or pneumothorax. If the procedure is the placement of a chest tube or an indwelling catheter, ultrasound can be used after drain insertion in order to identify the drain trajectory, excluding subcutaneous positioning (Figure 7). It is essential to highlight the fact that, because of air interposition, the intrapleural trajectory of the drain is seldom visible. However, there have been few studies assessing the results of standardized ultrasound-guided pleural drainage. In addition, to our knowledge, there have been no prospective studies comparing the complication rate of ultrasound-guided pleural drainage with that of conventional pleural drainage. Nevertheless, it is reasonable to assume that the use of ultrasound, by allowing a more detailed analysis of anatomy and offering a rapid means of identifying cases of subcutaneous positioning of the drain, would provide results that are more satisfactory than those provided by the conventional technique.^53^

### Ultrasound-guided thoracic biopsies

The technique of closed pleural biopsy to obtain diagnostic tissue has remained prevalent because of its ease of access and high level of acceptance among patients and medical professionals, particularly as an alternative to thoracoscopy and especially in regions with limited health care resources. There are no robust data to allow a distinction between a traditional (i.e., Cope or Abrams) reverse bevel and a core-cutting needle, in terms of specimen quality or diagnostic yield.^54^ ^,^ ^55^

Ultrasound can be a valuable tool for diagnosing undetermined thoracic lesions because it facilitates the collection of tissue from various structures such as the lung, chest wall, parietal pleura, and (anterior and upper) mediastinum.^54^ Ultrasound-guided biopsies can be performed whenever the use of ultrasound would allow a lesion to be visualized, which is not possible in many cases, such as in those of central lung tumors. Ultrasound-guided transthoracic needle biopsy is considered to have an acceptable diagnostic yield and is a cost-effective alternative to CT-guided biopsy,^55^ with a complication rate that is generally lower.^56^

### Vascular access

Vascular puncture is commonplace in ICUs. For over three decades, ultrasound has repeatedly been cited as an imaging method that can assist in vascular puncture. Currently, ultrasound-guided vascular puncture is part of the best practices for quality improvement and patient safety protocols.

The use of ultrasound guidance for vascular access is associated with a 60% reduction in complications such as pneumothorax and arterial punctures, as well as with a higher catheterization success rate.^57^ Ultrasound guidance provides several safety checkpoints. Ultrasound-guided vascular access uses a high-frequency linear probe, which provides detailed images of the superficial structures. The sonographer should identify the relevant structures, differentiate the vein from the artery, and exclude the presence of thrombi in the selected vessel before proceeding to venous puncture under real-time ultrasound guidance. Ultrasound can identify the guidewire position inside the venous structure, providing further safety before the dilation.

After the placement of an indwelling catheter, the sonographer can use ultrasound to evaluate the catheter position, mainly by one of two methods: by visualizing the tip of the catheter in the right atrium or vena cava, typically through a subcostal or right flank window,^58^ or by performing the bubble test, which is considered positive when a turbulent flow of intravenous fluid injected through the catheter is visualized in the right atrium or ventricle.^59^ Notably, the bubble test will ensure that the catheter is in the vascular system, even if the tip is not in the vena cava or the right atrium. Ultrasound confirmation of the catheter position reduces the need for X-rays and shortens the duration of catheter use for intravenous infusion.

## PERSPECTIVES

Medical utilization and ongoing research have solidified ultrasound as an essential, proficient tool for use in the modern clinic. Technological advances are enhancing the portability, accessibility, and cost-efficiency of ultrasound equipment. These characteristics allow the use of point-of-care ultrasound (POCUS) to extend beyond traditional hospital settings, reaching remote or resource-poor areas, ambulances, and clinics. In addition, the incorporation of artificial intelligence and machine learning algorithms has the potential to aid in interpreting images and recognizing patterns, potentially enhancing the capabilities of health care professionals. However, the increasing use of POCUS leads to ongoing deliberations about regulations and ethical considerations. Ensuring adequate training, standardization, patient confidentiality, data protection, and compliance with guidelines is crucial for its optimal, safe use. Therefore, specialized training programs are needed in order to equip health care professionals with the skills required for effective, accurate use of the technology. This training is pivotal to interpreting images accurately and maximizing the benefits of POCUS.

A well-designed, evidence-based curriculum for ultrasound training is imperative, akin to the requisites for clinical practice. Challenges such as a diverse caseload, inadequate specialized supervision, and different learning paces pose significant hurdles to education within a clinical setting. To ensure proficiency at each stage, appropriate and objective assessments, supported by robust validity evidence, are necessary before transitioning to independent practice. Overall, the future of POCUS appears promising as it continues to progress and integrate into diverse medical practices, providing real-time diagnostics and procedural support across a broad spectrum of health care settings.

## FINAL CONSIDERATIONS

Despite the initial low adoption rate, TUS is rapidly gaining traction as a diagnostic tool and a safety measure for interventional procedures in thoracic medicine. It is imperative to consider the TUS findings together with the clinical data for the correct interpretation of a diagnostic examination. It is not a question of TUS superiority over other diagnostic exams, such as X-ray and CT of the chest, but rather of the benefit of complementing the findings of other examinations and permitting point-of-care imaging.
